# Giant Gallbladder Tumor, Unusual Cancer—Case Report and Short Review of Literature

**DOI:** 10.3390/diagnostics13020194

**Published:** 2023-01-05

**Authors:** Adrian Constantin, Florin Achim, Tudor Turcu, Adelina Birceanu, Anca Evsei, Bogdan Socea, Dragos Predescu

**Affiliations:** 1 Department of Esophageal and General Surgery, “Saint Mary” Clinical Hospital Bucharest, 011192 Bucharest, Romania; 2 Faculty of Medicine, Carol Davila University of Medicine and Pharmacy Bucharest, 050474 Bucharest, Romania; 3 Department of Pathology, University Emergency Hospital Bucharest, 050098 Bucharest, Romania; 4 Pathoteam Diagnostic Bucharest Pathology Laboratory, 051923 Bucharest, Romania; 5 Department of Pathology, “Saint Mary” Clinical Hospital Bucharest, 011192 Bucharest, Romania; 6 Department of Surgery, Sf. Pantelimon Emergency Clinical Hospital, 021659 Bucharest, Romania

**Keywords:** cholecystectomy, intracholecystic papillary–tubular neoplasm, gallbladder cancer, giant gallbladder

## Abstract

Background: Giant gallbladder is an uncommon condition that can result from a benign pathology and rarely presents with malignancy. Intracholecystic papillary–tubular neoplasm (ICPN) is a relatively new entity first described by V. Adsay in 2012 and included in the World Health Classification of Digestive System Tumours in 2019. Intracholecystic papillary-tubular neoplasm is a preinvasive lesion with an incidence of around 1% that may present as four histologic subtypes—biliary, gastric, intestinal, or oncocytic—of which the biliary subtype has the highest risk of associated invasive cancer. Although invasive carcinoma is present in about 50% of cases of ICPN, these patients have a significantly better prognosis than those with usual gallbladder cancer, suggesting that the entities may have distinct biological signatures. Case report: A 77-year-old female presented to the hospital with progressive swelling in the right hemiabdomen, a loss of appetite, and weight loss. MRI highlighted a giant abdominal tumor located in the right hypochondrium and right abdominal flank with liver invasion (segment V). Preoperatively, a gallbladder 25 × 17 cm in size was noted, and the patient underwent radical cholecystectomy. It was surprising to find such a giant malignant gallbladder tumor, diagnosed as invasive poorly cohesive carcinoma associated with ICPN. Discussion: A megacholecyst is a rare discovery. Although most often found in benign pathologies, giant gallbladder cancer can be considered. The neoplastic features and the loco-regional extension of the tumor must be evaluated by imaging scans. Few cases of giant benign gallbladder have been reported in the literature; however, this appeared to be the largest resectable gallbladder carcinoma reported to date according to the literature. Conclusion: The stage of gallbladder neoplasia is not correlated with the size of the gallbladder. Regardless of tumor size, the prognosis seems to be directly related to the stage, morphology, and resectability.

## 1. Background

Bile duct cancer is a rare but aggressive neoplasm that includes intrahepatic cholangiocarcinoma; extrahepatic cholangiocarcinoma; and gallbladder cancer, which is the most common form with an incidence of 60–70% of cases [1]. At the same time, it is the fifth most common neoplasm of the gastrointestinal tract [2]. The incidence varies widely, with a peak in northern India, South America, and Pakistan [3,4]. Gallbladder adenocarcinomas have historically been classified into gland-forming (not otherwise specified), papillary, intestinal, pleomorphic giant cell, signet ring cell, mucinous, and clear cell types [5].

Intracholecystic papillary–tubular neoplasm (ICPN) was first described by V. Adsay, K-T Jang, et al. in 2012 [6] as a grossly visible, mass-forming, non-invasive epithelial neoplasm emerging in the mucosa and projecting into the lumen of the gallbladder, with a size ≥1 cm. Recognized by the World Health Organization in 2019 [7] as a preinvasive neoplasm of the gallbladder, ICPN shares many similarities with pancreatobiliary tract intraepithelial neoplasms, such as ITPN (intraductal tubulopapillary neoplasm), IPMN (intraductal papillary mucinous neoplasm), and IPN (intraductal papillary neoplasm of the bile ducts) and may exhibit four morphologically distinct patterns: the biliary, gastric, intestinal, or oncocytic phenotypes [6,8]. ICPN may be associated with an invasive carcinoma in more than 50% of cases, mainly pancreatobiliary type or other types, such as mucinous, adenosquamous, or neuroendocrine carcinomas, especially in cases with biliary phenotype and high-grade dysplasia [7,9,10].

Comparing ICPNs with or without subsequent invasive carcinoma and conventional gallbladder carcinoma, recent studies showed that patients with ICPN had a greater R0 resection rate; lower preoperative serum tumor markers (CEA, CA19-9); lower differentiation grades; lower rates of lymph node and distant metastases; and overall better prognosis, with a 5-year OS rate of 73.1% vs. 43.4% according to J. Seung Kang et al. and 60–78% vs. 18–30% according to Mochidome et al. [11,12]. The sequence of adenoma–dysplasia–invasive carcinoma has been described for gallbladder cancer but is found in several situations. In most cases, the degree of differentiation is weak [13]. Most of the time, elements of chronic inflammation are identified in the gallbladder wall and surrounding structures, which supports the hypothesis of the long evolution of the development from the stage of chronic inflammation to neoplasm. This interval is considered to be 9 years for men and 11 years for women [14]. Topographically, the neoplasm is located at the fundic level in 60% of situations, at the body level in 30%, and at the infundibular level in 10% [15]. The prognosis in the case of carcinoma alone is severe, with a 5-year survival rate of only 19%, being dependent almost exclusively on early diagnosis and the possibility of surgery [16]. Despite the possibility of curative treatment in the early stages, only 20% of cases are resectable at the time of diagnosis, and in 50% of cases lymph node metastases are detected [17,18].

Radical surgery is the mainstay of treatment. In over 60% of cases, however, there are relapses [19]. The standard first-line chemotherapy is the combination of gemcitabine–cisplatin for advanced cases with a median survival of less than one year [20]. Megacholecyst is an anatomo-clinical condition rarely described in certain benign pathologies (gallstones or chronic cholecystitis). It is known that the gallbladder mucocele can reach impressive sizes (up to the level of the iliac fossa). The detection of a giant malignant tumor at the level of the gallbladder is a completely exceptional situation that poses special problems for both the therapeutic approach and prognosis [21].

## 2. Case Presentation

A 77-year-old woman presented to the hospital with progressive swelling in the right hemiabdomen, a loss of appetite, and a weight loss of 8 kg in a period of 5 months. She was a former agricultural worker (including undocumented pesticide handling) and had no significant medical history, eight natural births, no alcohol or tobacco abuse, and no history of signs and symptoms of cholelithiasis. At the clinical examination, skin pallor and a firm, tender, slightly mobile lump in the right hypochondrium and the right iliac fossa, without signs of ascites, were detected.

The abdominal ultrasound revealed a solid mass occupying the entire right hemi-abdomen, though it could not provide details about the origin or the relationships with neighboring structures, due to the large size and the inhomogeneous character of the tumor.

**Figure 1:**
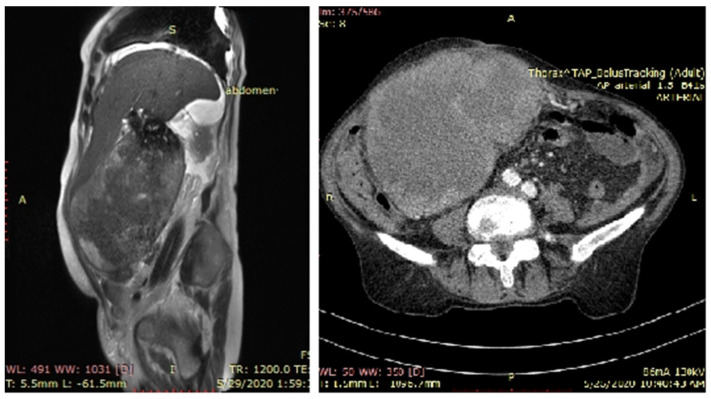
MRI (**left**) and CT (**right**) images, sagittal section and cross-section. A large subhepatic tumor mass occupied the entire flank and right hypochondrium, with the blurring of the demarcation line from the colon, duodenum, and jejunum, which appeared invaded by the tumor mass.

MRI highlighted an abdominal tumor located in the right hypochondrium and right abdominal flank with liver invasion (segment V). At the lower pole, the tumor wall was discontinuous. Intraperitoneal tumor extension was detected over a length of approximately 25 mm, with the invasion of the small bowel and omentum at this level. The tumor had a mixed appearance: solid-wall, gadolinophilic, with a maximum thickness of 35 mm and an inhomogeneous central liquid portion. Multiple stones, some of them with a diameter of 18 mm, were visible in the lower part of the tumor, but most agglutinated in the upper part, with dimensions varying between 5 and 30 mm. There were no lymph nodes and no metastasis. The common bile duct appeared with a normal caliber, alithiasic, and pushed antero-superior by the tumor mass, with no sign of invasion. This imaging result is characteristic of primitive gallbladder cancer (Figure 1). A cT3N0M0 stage was established under the limitations of the imaging evaluation difficulties regarding the large volume of the tumor, which made it hard to evaluate its relationships with the neighboring structures. CEA and CA 19.9 levels were within normal ranges.

**Figure 2:**
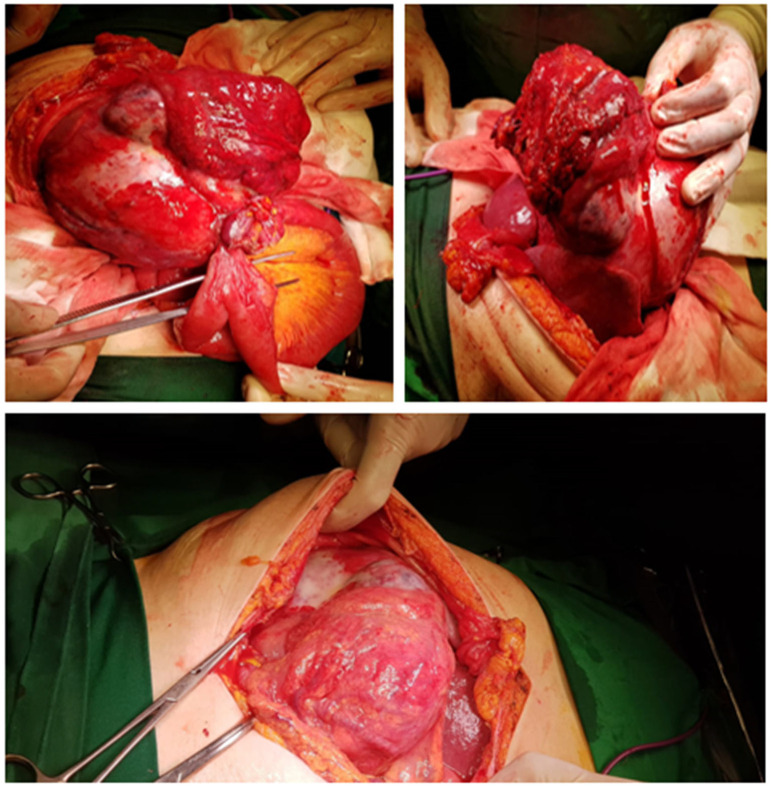
Intraoperative images. Gross appearance of a giant gallbladder tumor that presented adhesions with the neighboring structures, and aspects of peritonitis.

After the median laparotomy, a giant tumor (25/17 cm) occupying the entire right abdominal flank from the subhepatic region to the right iliac fossa was identified, which included the gallbladder, segment V of the liver, mesocolon, omentum, and a jejunal loop invasion, with numerous inflammatory adhesions with the neighboring structures (Figure 2). A cholecystectomy was performed with liver wedge resection, enterectomy, the partial resection of the mesocolon and omentum, and loco-regional lymphadenectomy. The complete resection of the tumor was performed en bloc with the abovementioned structures.

The postoperative evolution progressed without incident, with discharge on the 7th postoperative day.

The microscopic description was suggestive of biliary-type intracholecystic papillary–tubular neoplasm, with high-grade dysplasia/carcinoma in situ and without neoplastic invasion in neighboring structures, pT2aN0Mo.

The oncological (multidisciplinary) re-evaluation established an R0-type excision. Adjuvant treatment remained as a reserve (such as FOLFOX or FOLFIRI) in the case of loco-regional relapse or the appearance of metastases. Only clinical and paraclinical monitoring was decided.

The patient is still alive two and a half years later, with no signs of local recurrence or metastases.

**Figure 3:**
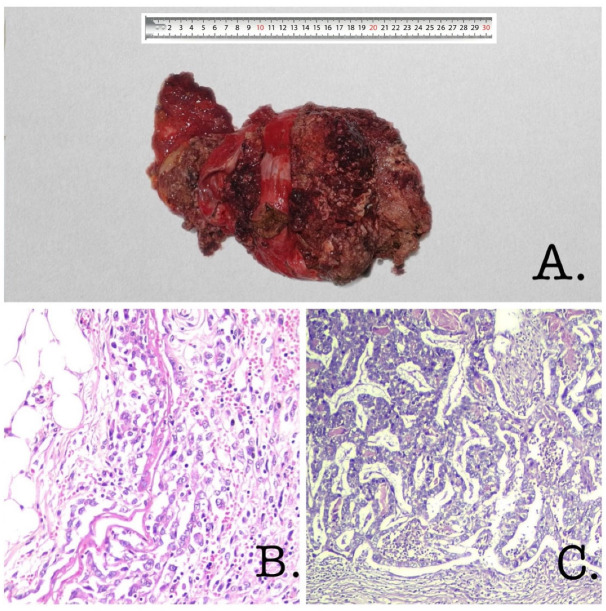
(**A**) Gross description: Nodular tumor with a size of 25/17/5 cm, irregular external surface, greyish-white color. Next to it, we identified a liver fragment with a size of 4.5/3/0.5 cm. The greater omentum was 14/8/1 cm in size, and the digestive tract segment was 3/3/1 cm. On section—mass with polypoid growth that was brittle, greyish-white, necrotic, protruding, and filling the entire lumen of the gallbladder. The tumor invaded the serosa of the gallbladder. Numerous yellow gallbladder stones were found inside the tumor, with hard consistency. (**B**) Hematoxylin and eosin ×200 (poorly cohesive cell carcinoma of the gallbladder): The histological study of the samples taken from the tumor showed the proliferation of carcinomatous cells with poorly cohesive cells and cord-like patterns forming a diffuse infiltrative growth invading the fat tissue. Neoplastic cells were round or oval, irregular in shape, with moderate cytonuclear atypia. The nuclei were vesicular and the nucleoli were eosinophilic and visible. The microscopic description suggested poorly cohesive cell carcinoma of the gallbladder. (**C**) Hematoxylin and eosin ×100 (intracholecystic papillary–tubular neoplasm of the gallbladder): The histological study of the samples taken from the neoplasm showed back-to-back epithelial units with a tubulopapilary growth pattern and limited stroma. The papillae were lined with cuboidal cells with eosinophilic cytoplasms that were enlarged, pleomorphic, and had atypical nuclei with distinct nucleoli; architectural complexity and a loss of polarity were also observed. The microscopic description suggested intracholecystic papillary–tubular neoplasm of the biliary type with high-grade dysplasia/carcinoma in situ.

The case depicted in Figure 3 exhibited a poorly differentiated adenocarcinoma with a poorly cohesive cell pattern (Figure 3B), associated with a pancreatobiliary-type ICPN with high-grade dysplasia (Figure 3C), being diffusely positive for specific lineage markers such as CK7 and MUC1 and expressing focal MUC5AC and MUC6.

## 3. Discussion

Gallbladder cancer accounts for 1.2% of all neoplasms and 1.7% of neoplasm deaths. According to Global Cancer Statistics (GLOBOCAN) data from 2018, it ranks 22nd in terms of incidence and 17th in terms of aggression. It is 2–6 times more common in women [22]. The highest incidence is reported in South American countries such as Chile and Bolivia (it is the leading cause of cancer-related death in women in Chile), followed by India, Central Europe, Japan, and Israel [23].

There is an annual incidence of 1.2–3/100,000 in the USA, with an estimated 10,310 new cases and 3230 deaths in 2013 [24]. It is known that early diagnosis and surgical treatment (before the invasion of the mucosa) leads to a 5-year survival rate of almost 100% even without adjuvant treatment and less than 15% long-term survival in the event of the invasion of the muscular layer [25]. Due to its very thin lamina propria and muscular layer, the cancer quickly invades both the lymphatic network and the neighboring structures. The terminal stages are characterized by peritoneal spreading, malignant ascites, and liver and lung metastases. Breast metastases have been described [26].

The discouraging results are also justified by the resistance to radiotherapy and the usual chemotherapies, as well as by the surgical difficulty regarding a radical excision. Risk factors include gallstones (present in 85% of patients with gallbladder cancer). The risk of malignancy is 2.4 times higher in patients with stones between 2.0 and 2.9 cm compared to those with stones under 1 cm. This risk increases 10-fold for stones over 3 cm. Additionally, the risk of malignancy increases with the time of evolution, being almost 5 times higher for an evolution of 5–19 years and over 6 times higher for over 20 years [27]. Other risk factors are a porcelain gallbladder, primitive sclerosing cholangitis, biliary-pancreatic junction abnormalities, polyps over 10mm, a solitary polyp, sessile polyps, polyps associated with gallstones, and polyps in patients over 65 years [28]. Old age, female sex, alcohol and tobacco abuse, Helicobacter infection, multiple pregnancies, a sedentary lifestyle, and obesity are implicated in the occurrence of gallbladder cancer in all ethnic groups [29]. It is difficult to identify a relationship between gallbladder neoplasm and genetic issues due to the low incidence of the disease [6].

In the USA, the survival rate for stages II, III, and IV are 49, 24, and 8 months, respectively [30]. The symptoms are very vague compared to the rapid progression of the disease, which contributes to the diagnosis at advanced stages and the unfavorable prognosis at the time of diagnosis [31]. In most patients, only moderate sensitivity in the right hypochondrium is detected, or the disease presents via accidental imaging discoveries or histological surprises. Cholecyst neoplasm is detected in approximately 0.5–1.5% of cholecystectomy specimens for gallstones [32]. The presence of jaundice, abdominal lumps, anorexia, or weight loss are signs of advanced stages. Additionally, a gallbladder mucocele in the absence of lithiasis may be an indirect sign of neoplasia in the cystic duct or infundibulum [33]. Gallbladder cancer is rarely diagnosed before it is locally advanced or in a metastatic stage [34].

Early-stage diagnosis is important for improving prognosis. Ultrasound, CT, and MRI are essential in establishing diagnoses and treatment protocols. The presence of an intracolecystic tissue mass is evident in 40–65% of patients. Gallbladder cancer may present as an asymmetric thickening of the gallbladder wall that can be seen via CT with contrast or MRI [35]. Suggestive features for gallbladder cancer are the presence of an intracholecystic tissue mass, the focal thickening of the gallbladder wall, the “double contour” appearance of the gallbladder wall, the infiltration of surrounding structures, the presence of loco-regional lymphadenopathy, and liver or intraperitoneal metastases [36]. The diffuse thickening of the gallbladder wall may suggest a benign pathology, while asymmetric, irregular, or extensive modification indicates neoplasia [31].

Radical surgery is the mainstay of gallbladder cancer treatment and involves cholecystectomy with 3 cm of liver tissue in segments IVB and V and loco-regional lymphadenectomy. A minimum of six lymph nodes are required for proper staging [37]. Staging laparoscopy can prevent unnecessary laparotomy in 27.6% of cases in patients with gallbladder cancer [38]. After a systematic review performed by Gupta et al., it was shown that the possibility of an R0 resection after neoadjuvant chemotherapy could increase by 15–86% at stages T3 and T4 [39]. Adjuvant chemotherapy with 5-fluorouracil and gemcitabine has been reported to have important benefits in patients with positive post-resection margins, lymph node metastases, or stages T3 and T4 [40].

Preclinical and clinical studies have shown that nab-paclitaxel increases the intratumoral concentration of gemcitabine by decreasing the cytidine deaminase (gemcitabine metabolizing enzyme) levels [41,42]. Due to the synergistic antitumor effect of nab-paclitaxel and gemcitabine, this combination has been shown to be effective for advanced pancreatic cancer as a first-line treatment [43]. The subsequent combination of nab-paclitaxel with gemcitabine–cisplatin therapy has been shown to prolong disease-free interval and survival in cases of advanced biliary tract neoplasm [44]. Effective tumor markers are not available for the surveillance of these cases, which are known to be prone to relapses and metastasis [1]. Although there are no clear-cut definitions, gallbladders of size > 14 cm and volume ≥ 1.5 L have been regarded as giant gallbladders.

Resectable malignant gallbladders of such a size are rare (Table 1).

The small number of reported cases does not allow the issuance of pertinent hypotheses regarding the mechanism of development of giant neoplasms at this level. It can only be speculated that aggressive local inflammatory phenomena are involved, possibly associated with a low level of oncological aggression that is difficult to quantify. From the point of view of surgical treatment, it is known that laparoscopic cholecystectomy for megacholecysts with benign pathology can be performed in centers with appropriate experience [22,48]. Laparoscopic radical cholecystectomy can be performed in centers with experience for stage T1 and T2 gallbladder cancer with satisfactory results [49,50]. However, open radical cholecystectomy is the current standard, especially in the case of a megacholecyst [4].

## 4. Conclusions

A megacholecyst is a rare discovery. Although most often found in benign pathologies, giant gallbladder cancer can be considered. The neoplastic features and the loco-regional extension of the tumor must be evaluated by imaging scans. In the case of a giant tumor, this becomes a real challenge. If the neoplastic characteristics are relatively easily identifiable (thickened, inhomogeneous, irregular wall), the invasion of the surrounding structures becomes a real challenge due to the large tumor size, consecutive inflammatory phenomena, and natural relationships between the gallbladder and important structures. On the other hand, the stage of gallbladder neoplasia is not correlated with the size of the gallbladder. At the same time, the prognosis seems to be directly related to the stage and resectability, regardless of size. Herein, the identification of a gallbladder neoplasm of impressive size did not necessarily support the decision to abstain from surgery. Due to the limited number of cases, it can only be speculated that the causes of these exceptional situations are related to aggressive inflammatory processes, with a long evolution correlated to a particularly low oncological aggression, a factor that is difficult to quantify. Another logical scenario could be the development of a biliary carcinoma on a pre-existing megacholecyst secondary to a benign condition (intracholecystic tubule–papillary neoplasm), but none of the reported cases (including our case) were documented before the development of the neoplasm in such a condition. Possible factors associated with the invasiveness are papilla formation, cell type (biliary or foveolar), and high-grade dysplasia.

## Figures and Tables

**Table 1:** Giant malignant gall bladders reported to date.

Article	Age (Years)	Sex	GB Size (cm)	GB Volume	Diagnosis
1. Bains, Maranna et al., 2020 [4]	65	F	24 × 9	NR	Gall bladder adenocarcinoma
2. Chapman et al. [5]	59	F	18	NR	Gall bladder adenocarcinoma with liver metastasis
3. Hsu et al. [45]	87	F	16.4 × 13.6 × 7.8	NR	Gall bladder adenocarcinoma with empyema
4. Sofia Pina et al. [46]	61	M	20	NR	Gall bladder adenocarcinoma 2021
5. Junior MAR et al. [47]	45	M	NR	NR	Gall bladder squamous cell carcinoma

## Data Availability

The data presented in this study are available on request from the corresponding author.
